# Supplementary figures and images for: Impacts of Habitual Diets Intake on Gut Microbial Counts in Healthy Japanese Adults
Source: Nutrients. 2020 Aug 12;12(8):2414. doi: 10.3390/nu12082414 (PMC7468936; doi:10.3390/nu12082414)

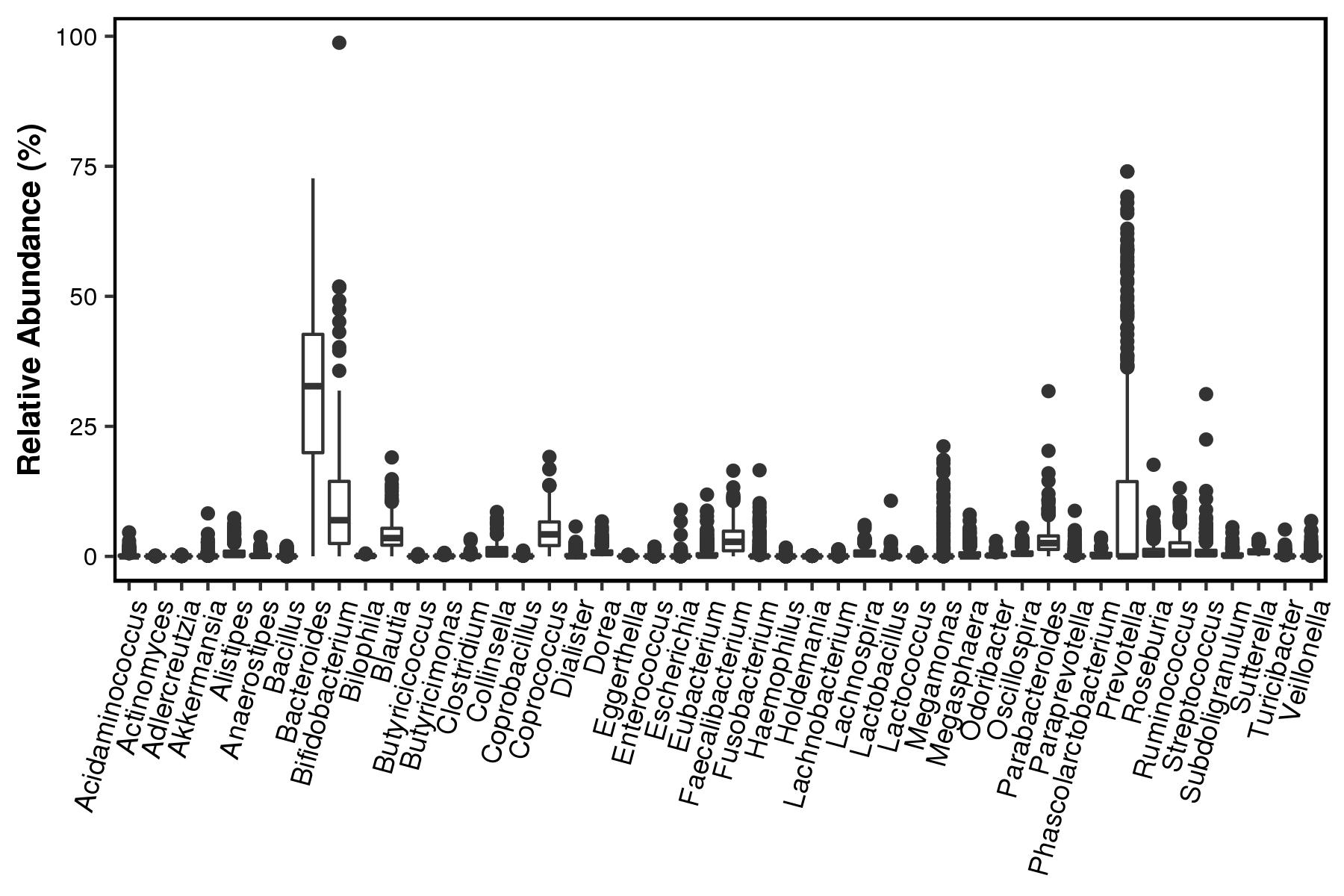

Supplement: Supplementary file 1 [file nutrients-12-02414-s001.zip › Supplementary file/supplementary figure 1.jpeg]
